# Supplementary material for: Self-care practices and associated factors among type 2 diabetes mellitus patients attending public hospitals in Bale zone, Oromia region, Ethiopia
Source: PeerJ. 2025 Jun 20;13:e19529. doi: 10.7717/peerj.19529 (PMC12184674; doi:10.7717/peerj.19529)
Supplement: Supplemental Information 4 [file peerj-13-19529-s004.pdf]

**Self-Care Practices and Associated Factors Among Type 2 Diabetes Mellitus Patients Attending Public Hospitals in Bale Zone, Oromia Region, Ethiopia.**

## Questionnaire

**# Unique Number________________**

Questionnaire (**English Version**)

**PART I:-** Socio-Demographic Characteristics

| S.no | Questions | Possible response | Remark |
|---|---|---|---|
| Q101 | Age (in years) | __________years | |
| Q102 | Sex | Male<br>2. Female | |
| Q103 | Marital Status | Single<br>2. Married<br>3. Divorced<br>4. Widowed | |
| Q104 | Educational Status | 1. Not able to read and write<br>2. Able to read and write only<br>3. Primary education /1-8/<br>4. Secondary education /9-12/<br>5. Above grade 12 | |
| Q105 | Occupation | 1. Governmental employee<br>2. Private employee<br>3. Merchant<br>4. Housewife<br>5. Farmer<br>6. Daily laborer<br>7. Student<br>8. Other Specify | |
| Q106 | Economic status(monthly income) | --------------- birr | |
| **PART II** –Clinical and other factors related to diabetes | | | |

| No | Questions | Possible responses | Remark |
|---|---|---|---|
| Q201 | How long you have been diagnosed with diabetes Mellitus | ----------------------years | |
| Q202 | Do you have any diabetic-related long-term complications? | Yes<br>No | |
| Q203 | If your answer is yes Q202 which type of complications do have? (multiple answers are possible) | Diabetic nephropathy<br>Diabetic neuropathy<br>Diabetic retinopathy<br>Diabetic foot ulcer<br>Diabetic-related heart disease<br>Others(specify) | |
| Q204 | Have you ever diabetic health education about diabetes self-care | Yes<br>No | |
| Q205 | If your answer is yes to Q204 from where did you set health education | Doctors(others health personal)<br>Mass media<br>Friends or family<br>Others(specify) | |
| Q206 | Are you a member of the diabetic association | Yes<br>No | |

**PART III** – Self-care Knowledge Questionnaire

Modifiable lifestyle (standard..Diabetes knowledge test questionnaire 18 items)

| No | Questions | Possible responses | | Remark |
|---|---|---|---|---|
| | | Yes | No | |
| Q301 | Are you aware of the importance of maintaining a balanced diet for overall health and wellness? | | | |
| Q302 | Do you understand the significance of regular physical activity in improving your lifestyle? | | | |
| Q303 | Are you knowledgeable about the adverse effects of smoking and the benefits of quitting? | | | |
| Q304 | Do you know the recommended guidelines for alcohol consumption and the potential risks of excessive drinking? | | | |
| Q305 | Are you aware of the impact of stress on your health and the importance of stress management techniques? | | | |
| Q306 | Do you understand the importance of adequate sleep for your overall well-being? | | | |
| Q307 | Are you knowledgeable about the benefits of maintaining a healthy body weight? | | | |
| Q308 | Do you understand the risks associated with excessive sedentary behaviour and the importance of regular movement throughout the day? | | | |
| Q309 | Are you aware of the significance of maintaining good oral hygiene for overall health? | | | |
| Q310 | Do you understand the importance of regular health check-ups and screenings for early detection of potential health issues? | | | |
| Q311 | Are you knowledgeable about the harmful effects of prolonged exposure to the sun and the importance of using sun protection? | | | |
| Q312 | Do you understand the benefits of drinking an adequate amount of water and staying hydrated? | | | |
| Q313 | Are you aware of the potential risks of excessive consumption of processed foods and the benefits of choosing whole, unprocessed foods? | | | |
| Q314 | Do you understand the impact of excessive caffeine intake | | | |

| | | | | |
|---|---|---|---|---|
| | on your health and the importance of moderation? | | | |
| Q315 | Are you knowledgeable about the benefits of maintaining social connections and engaging in positive relationships? | | | |
| Q316 | Do you know the importance of allocating time for relaxation and engaging in hobbies or activities you enjoy? | | | |
| Q317 | Are you aware of the impact of excessive screen time on your physical and mental well-being? | | | |
| Q318 | Do you understand the benefits of practising mindfulness and incorporating stress-reduction techniques into your daily life? | | | |

**Medication Adherence** (8 risky item tool )

| No | Questions | Possible responses | | Remark |
|---|---|---|---|---|
| | | Yes | No | |
| Q319 | Do you sometimes forget to take your diabetes pills? | | | |
| Q320 | People sometimes miss taking their medications for reasons other than forgetting. Thinking over the past two weeks, were there any days when you did not take your medicine? | | | |
| Q321 | Have you ever cut back or stopped taking your medicine without telling your doctor because you felt worse when you took it? | | | |
| Q322 | Are you sometimes when you travel or leave home, in a hurry and forget to take your diabetes medication? | | | |
| Q323 | . Did you take all your medicine yesterday? | | | |
| Q324 | When you feel like your symptoms are under control do you sometimes stop taking your medicine? | | | |
| Q325 | Taking medicine every day is a real inconvenience for some people, do you ever feel hassled about sticking to your treatment plan? | | | |
| Q326 | How often do you have difficulty remembering to take all your medicine? | | | |
| **A consequence of uncontrolled blood sugar level** (4 items) | | | | |
| Q327 | If blood sugar is close to normal, a person with diabetes is likely to have more energy, feel less thirsty, and urinate less often | | | |
| Q328 | Prolonged high blood sugar levels can cause eye problems or even blindness | | | |
| Q329 | Prolonged uncontrolled blood sugar levels can cause heart attack, stroke, and kidney problem | | | |

| | | | | |
|---|---|---|---|---|
| Q330 | Shaking, confusion, behavioural change, and sweating are signs of high blood sugar | | | |

**PART IV:** SUMMARY OF DIABETES SELF-CARE ACTIVITIES QUESTIONNAIRE

The questions below ask you about your diabetes self-care activities during the past 7 days. If you were sick during the past 7 days, please think back to the last 7 days that you were not sick.

**Diet**

| S.no | Questions | Possible response | Remark |
|---|---|---|---|
| Q401 | How many of the last seven days have you followed a healthy day's eating plan? | ------Number of Days | |
| Q402 | On average, over the past month, how many DAYS PER WEEK have you followed you're eating plan? | ------Number of Days | |
| Q403 | On how many of the last SEVEN DAYS did you eat five or more servings of fruits and vegetables? | ------Number of Days | |
| Q404 | On how many of the last seven days did you eat high-fat foods, such as red meat or full-fat dairy products? | -----Number of Days | |
| Q405 | On how many of the last SEVEN DAYS did you space carbohydrates evenly through the day? | -----Number of Days | |

**Physical Activity**

| S.no | Questions | Possible response | Remark |
|---|---|---|---|
| Q406 | On how many of the last seven days did you participate in at least 30 minutes of physical activity (total minutes of continuous activity, including walking)? | ---------------Number of Days | |
| Q407 | On how many of the last seven days did you participate in a specific exercise session (such as swimming, walking, or working) other than what you do around the house or as part of your work? | ---------------Number of Days | |

**Blood Sugar Testing**

| S.no | Questions | Possible response | Remark |
|---|---|---|---|
| Q408 | On how many of the last seven days did you test your blood sugar? | ---------------Number of Days | |
| Q409 | On how many of the last seven days did you test your blood sugar the number of times recommended | ---------------Number of Days | |

| | | | |
|---|---|---|---|
| | by your healthcare provider? | | |

**Foot Care**

| S.no | Questions | Possible response | Remark |
|---|---|---|---|
| Q410 | On how many of the last SEVEN DAYS did you check your feet? | -----------Number of Days | |
| Q411 | On how many of the last seven days did you inspect the inside of your shoes? | ----------Number of Days | |
| Q412 | On how many of the last seven days did you wash your feet? | -----------Number of Days | |
| Q413 | On how many of the last seven days did you soak your feet? | ---------Number of Days | |
| Q414 | On how many of the last seven days did you dry between your toes after washing? | ----------Number of Days | |

**Medication**

| **S.no** | **Questions** | **Possible response** | **Remark** |
|---|---|---|---|
| Q415 | On how many of the last seven days did you take your recommended diabetes medication? | | |
| Q416 | On how many of the last seven days did you take your recommended insulin injections? | | |
| Q417 | On how many of the last seven days did you take your recommended number of diabetes pills? | | |

**Smoking**

| S.no | Questions | Possible response | Remark |
|---|---|---|---|
| Q418 | Have you smoked a cigarette, even a puff, in the past seven days? | Yes<br>No | |

**PART V**: Self-Care Recommendations (Health Professionals Advice)

**DIET MANAGEMENT seven**

Which of the following has your healthcare team (doctor, nurse, dietitian, or diabetes educator) advised you to do? Please check all that apply.

| S.no | Recommendations (HQuestions | Possible response | | Remark |
|---|---|---|---|---|
| | | Yes | No | |

| | | | | |
|---|---|---|---|---|
| 1.1 | Follow a low-fat eating plan | | | |
| 1.2 | Follow a complex carbohydrate diet | | | |
| 1.3 | Reduce the number of calories you eat to lose weight | | | |
| 1.4 | Eat lots of food high in dietary fibre | | | |
| 1.5 | Eat lots (at least 5 servings per day) of fruits and vegetables | | | |
| 1.6 | Eat very few sweets (for example, desserts, non-diet sodas, and candy bars) | | | |
| 1.7 | I have not been given any advice about my diet by my healthcare team | | | |

**PHYSICAL EXERCISE:** Which of the following has your healthcare team (doctor, nurse, dietitian, or diabetes educator) advised you to do? Please check all that apply.

| S.no | Questions | Possible response | | Remark |
|---|---|---|---|---|
| | | Yes | No | |
| 2.1 | Get low-level exercise (such as walking) daily | | | |
| 2.2 | Exercise continuously for at least 20 minutes, at least 3 times a week | | | |
| 2.3 | Fit exercise into your daily routine (for example, take stairs instead of elevators, park a block away and walk, etc.) | | | |
| 2.4 | Engage in a specific amount, type, duration, and level of exercise | | | |
| 2.5 | I have not been given any advice about exercise by my healthcare team | | | |

**SUGAR LEVEL:** Which of the following has your healthcare team (doctor, nurse, dietitian, or diabetes educator) advised you to do? Please check all that apply

| S.no | Questions | Possible response | | Remark |
|---|---|---|---|---|
| | | Yes | No | |
| 3.1 | Test your blood sugar using a drop of blood from your finger and a color chart | | | |
| 3.2 | Test your blood sugar using a machine to read the results | | | |
| 3.3 | Test your urine for sugar | | | |

| | | | | |
|---|---|---|---|---|
| 3.4 | I have not been given any advice about my blood or urine sugar level by my healthcare team | | | |

**SMOKING I have**

| S.no | Questions | Possible response | | Remark |
|---|---|---|---|---|
| | | Yes | No | |
| 4.1 | At your last doctor's visit, did anyone ask about your smoking status? | | | |
| 4.2 | If you smoke, at your last doctor's visit, did anyone counsel you about stopping smoking? | | | |
| 4.3 | Do not smoke. (Never smoke.) | | | |
| 4.4 | When did you last smoke a cigarette? never smoked | | | |
| 4.5 | smoke. When did you last smoke a cigarette? More than two years ago | | | |
| 4.6 | When did you last smoke a cigarette? One to two years ago | | | |
| 4.7 | When did you last smoke a cigarette? Four to twelve months ago | | | |
| 4.8 | When did you last smoke a cigarette? One to three months ago | | | |
| 4.9 | When did you last smoke a cigarette? Within the last month | | | |
| 4.10 | When did you last smoke a cigarette? Today | | | |

I have completed the questions. Thank you very much!!!
