# Supplementary material for: Self-care practices and associated factors among type 2 diabetes mellitus patients attending public hospitals in Bale zone, Oromia region, Ethiopia
Source: PeerJ. 2025 Jun 20;13:e19529. doi: 10.7717/peerj.19529 (PMC12184674; doi:10.7717/peerj.19529)
Supplement: Supplemental Information 5 [file peerj-13-19529-s005.pdf]

GAAFANNOO (AFAN OROMO VERSION)

Code No___________________

Qorannoon kun dhukkubsattoota bdhibee sukkaaraa gosa 2ffaa umrii Waggaa 18 olii ilaallata

- **Cheekliistiin kun kutaa gurguddaa 5 kan qabu yoo tahu ,gaaffilee asirratti tarreeffaman of -eeggannoon deebii sirrii akka nuuf guuttan kabajaa guddaan isin hubachiifna.Yeroo keessan Aarsaa gootanii ragaa kana guutuu keessaniif ulfinaaf kabajaa isiniif qabna**

KUTAA I-SOCIO-DEMOGRAPHIC CHARACTERISTICS

T.L	**Gaafannoo**	**Deebii kennamuu dandaú**	**Yaada**
Q101	Umrii (in years)	__________Waggaan	
Q102	Saala	Dhi Du	
Q103	Haala Gaaílaa	1. Qeenxee<br>2. Kan fuudhe/te<br>3. kan hiik/te<br>4. 'Dubartii haadha Warraa taate	
Q104	Haala Barnootaa	1. Kan Barreessuu fi dubbisuu hin dandeenye<br>2. Barreessuu fi dubbisuu kan dandahu<br>3. Barnoota sadarkaa duraa /1-8/<br>4. Barnoota Sadarkaa 2ffaa<br>/9-12/<br>5. Daree 12 ol kan barate	
Q105	Hojii	1. Hojjataa Mootummaa<br>2. Hojii Dhuuunfaa<br>3. Daldaalaa<br>4. Haadha Mana<br>5. Qote Bulaa<br>6. Dafqaan Bulaa<br>7. Barataa<br>8. Kan biroo ibsi	
Q106	Haala Galii(monthly income)	--------------- birr	

KUTAA 2 –WANTOOTA DHIBEE SUKKAARAA WAJJIIN WALQABATEE FI SABABOOTA BIROO

No	Gaafannoo	Deebii tahuu kan dandahu	Yaada
Q201	Qorannoo dhibee Sukkaaraa erga jalqabdanii yeroo hagamii taha?	-----------------------Waggaadhaan	

Q202	Dhibee Sukkaaratiin walqabatee wanti isinitti dabalatan dhufe jiraa?	Eeyyee<br>Lakkii	
Q203	Yoo gaaffii Q202 deebii isaa eeyyee jettan, kanneen keessaa 1. Sababa Dhibee Sukkaaraan kan dhufe kamtu isinirratti muldhate? (Deebii filannoo heddu deebisuun ni dandaáma)	kalee Dhukkuba Sukkaaraa<br>Narvii Dhukkuba Sukkaaraa<br>Dhibee ijaa<br>Madaaúu Miilaa<br>Dhibee onnee<br>Kan biroo(ibsaa)	
Q204	Waa'ee dhibee sukkaaraa fi haala fayyaa keessanii irratti barnootni fayyaa isiniif kennamee beekaa?	Eeyyee<br>Lakkii	
Q205	Gaaffii Q204 deebiin keessan eeyyee yoo tahe,barnoota fayyaa eessatti argattan?	Doctors(Ogeeyyii fayyaa biroo)<br>Miidiyarraa<br>Hiriyaa ykn Maatii irraa<br>Kan Biroo(Ibsi)	
Q206	Ati Waldaa Miseensota Dhibee Sukkaaraa keessa jirtuu?	Eeyyee<br>Lakkii	

KUTAA 3 – Gaafannoo Beekumsa Hubannoo Fayyaa ofii eeggachuu irratti Qaban

Akkaataa Haala jireenyaa Fooyyessuu dandahamu irratti (18 items)

T.L	Gaafannoo	Deebii kan tahuu dandahu		Yaada
		Eeyyee	Lakkii	
Q301	Sochii Qaamaa hundi taasifamu jalqabaa fi booda irratti sadarkaan gulukosii dhiigaa safaramuu qaba			
Q302	Qorannoo Sukkaara dhiigaa ariitii(FBT) fayyadamuun tooánnoo sukkaara dhiigaa jiá 2-3 hordofuun ni dandaáma.			
Q303	Namni dhukkuba sukkaaraa qabu ilkaan isaa buruushiin dhiqachuu fi ilkaan isaa/floos gochuu qaba.			
Q304	Hoosii cimaan ykn kaalsiin nama dhukkuba sukkaaraa qabuuf hamaa miti.			
Q305	Namni dhukkuba sukkaaraa qabu jijjiirama sadarkaa sukkaara dhiiga isaa hordofuu fi deebii akka kennu isa dandeessisa			
Q306	Namni dhukkuba sukkaaraa qbu tokko akkamitti galma kaayyeffate galmaan gahuu akka dandahu karoora baasuu kan qabu hakiima qofa			
Q307	Ulfaatina qaama fayya qabeessa qabaachuun bulchiinsa dhukkuba sukkaaraa keessatti barbaachisaa miti			
Q308	Hordoffiigilukoosii dhiigaa ofumaan(SBGM)Doktoroonnii fi gareewwan			

	eegumsa fayyaa biroo yaalaaf raga akka walitti qaban taasisa			
Q309	Doktoroota fayyaa gahumsa qabanii fi hojjettoota fayyaa biroo hospitaala keessa jiran malee Namni kamiiyyuu Sukkaara dhiigaa fi dhiibbaa dhiigaa ilaaluu hin qabu.			
Q310	Sagantaa tokkotti daqiiqaa 20-30f sochii qaamaa yoo xiqqaate torbanitti guyyaa sadi qabaachuun fakkeenya barbaachisaa sochii qaamati.fkn:Deemsa Ariifata,Sochii manaa,Saayikilii oofuu,sadarkaa ol bahuu			
Q311	Namni dhukkuba sukkaaraa qabu jijjiirama ija isaa hunda doktora isaaf gabaasuu qaba.			
Q312	Namni dhukkuba sukkaaraa qabuu fi hakiimota gidduutti waliigaltee waliinii jiraachuu qaba/yoo inni ykn isheen akka akkaataa jireenya murtaaé jijjiiruu hin dandeenye			
Q313	Namni dhukkuba Sukkaaraa qabu keessumaa yeroo cirrachi miila isaa muru ,miila isaa/ishee kunuunsuu dabaluu qaba.			
Q314	Sochii Qaamaa yeroo hunda gochuun barbaachisummaa insulinii ykn Qoricha dhukkuba sukkaara biroo hirísa			
Q315	Namni dhukkuba sukkaaraa qabu tokko garee eegumsa fayyaa isaa irraa yeroo dhukkubni itti dhagahamu qofa gargaarsa gaafachuu qaba.			
Q316	Jalqaba yaala insuliinii nama dhukkuba sukkaraa qabuuf gorsa sirrii ykn hordoffii gilukoosii dhiiga ofii(SBGM) fi nyata nama sanaaf kennamuu qaba..			
Q317	Sigaaraa xuuxuun dhukkuba sukkaaraa hammeessuu dandaha			
Q318	Dhiibbaa dhiigaa hordofuun akka nama dhukkuba sukkaaraa qabu keessatti gilukoosii dhiigaa hordofuu barbaachisaa miti..			

Abboomamuu (8 items)

lakk	Gaaffilee	Deebii kennamuu dandaú		Yaada
		Eeyyee	Lakki	
Q319	Yeroo tokko tokko kiniinii dhukkuba sukkaaraa fudhachuu ni dagattaa?			
Q320	Namoonni yeroo tokko tokko qoricha isaanii fudhachuu irraanfatamuu malee sababa biraatiin ni hafu .Torban lamaan darban keessa yaaduun ,guyyoonni qoricha kee hin fudhanne jiraa?			
Q321	Qoricha kee yeroo fudhattu sitti hammaate waan ta’eef osoo hakiima keetti hin himiin fudhachuu kee hir’istee ykn dhiiftee beektaa?			
Q322	Yeroo tokko tokko yeroo imala ykn manaa baatu , ariifattee qoricha dhukkuba sukkaaraa fudhachuu dagattu?			
Q323	Kaleessa qoricha kee hunda fudhattee?			
Q324	Mallattoon kee to'annaa jala akka jiru yeroo sitti dhaga'amu yeroo tokko tokko qoricha kee fudhachuu ni dhiistaa ?			
Q325	Guyyaa guyyaan qoricha fudhachuun namoota tokko tokkoof rakkina dhugaati,karoora wal'aansa kee irratti maxxanuu irratti rakkinni sitti dhaga'amee beekaa?			

Q326	Yeroo meeqa qoricha kee hunda fudhachuu yaadachuu sitti ulfaata?			

Buáa Sadarkaa Sukkaarri Dhiigaa tooánnoon ala taúu (4 items)

Q327	Sukkaarri dhiigaa yeroo idileetti yoo dhiyaate namni dhukkuba sukkaaraa qabu human guddaa qabaachuu,dheebuun isaa xiqqaachuu fi fincaan baayísuun isaa yeroo xiqqaadhaf			
Q328	Yeroo dheeraaf hammi sukkaara dhiigaa olkaúun rakkoo ykn ija jaamsuu ille fiduu dandaá.			
Q329	Yeroo dheeraaf hammi sukkaara dhiigaa tooánnaa ala taúun dhukkuba onnee ,dhiigni sammuu keessatti dhangalaúu fi rakkoo tiruu fiduu dandaá.			
Q330	Mallattooleen Sukkaara dhiigaa olkaúu Raafamni,burjaajjaúun,Jijjiiramni amalaa fi ‘dafqaa			

KUTAA AFUR: CUUNFAA GAAFFILEE HOJII OF EEGGANNOO DHUKKUBA SUKKAARAA

Gaaffiiwwan Armaan gadii waa’ee sochii of kunuunsuu dhukkuba sukkaaraa guyyoota 7 darban keessatti goote si gaafatu.Guyyoota 7 darban keessa yoo dhukkubsattee turte ,mee guyyoota 7 darban kan hin dhukkubsanne sanatti deebi’ii yaadi.

Nyaata

T.lakk	Gaaffilee	Deebii tahuu dandahu	Yaada
Q401	Guyyoota 7 darban keessattikaroora nyaata fayya qabeessa guyyaa meeqaaf hordofte?	---------------lakkoofsa guyyootaa	
Q402	Giddugaleessaan,ji’a darbe keessa,torban keessatti guyyaa meeqa karoora nyaata nyaachaa jirtu hordofteetta?	---------------lakkoofsa guyyootaa	
Q403	Guyyoota torban darban keessaa,guyyoota meeqaaf kuduraa fi muduraa shanii fi isaa ol nyaatte?	---------------lakkoofsa guyyootaa	
Q404	Guyyoota torban darban keessaa guyyaa meeqaaf ,nyaata cooma baay’ee qabu kan akka foon diimaa yookiin oomishaalee aannanii cooma guutuu qaban nyaatte?	---------------lakkoofsa guyyootaa	
Q405	Guyyoota torban darban keessaa guyyaa meeqaaf,nyaata kaata kaarboohydreetii walqixa addaan baafatte?	---------------lakkoofsa guyyootaa	

Sochii Qaamaa

T.lakk	Gaaffilee	Deebii tahuu dandahu	Yaada
Q406	Guyyoota torban darban keessaa guyyaa meeqaaf yoo xiqqaate daqiiqaa 30’f sochii qaamaa(fkn sochii walitti fufiinsa qabu,deemsadabalatee) irratti hirmaatte?	---------------lakkoofsa guyyootaa	
Q407	Guyyoota torban darban keessaa guyyaa meeqaaf waan naannoo mana keetti yookiin akka qaama hojii keetti hojjattu malee (kan akka deemsa,biskileetii oofuu) irratti hirmaatte?	---------------lakkoofsa guyyootaa	

Qorannoo Sukkaara Dhiigaa

T.lakk	Gaaffilee	Deebii tahuu dandahu	Yaada
Q408	Guyyoota torban darban keessaa guyyaa meeqaaf,sukkaara dhiiga kee qoratte?	---------------lakkoofsa guyyootaa	

Q409	Guyyoota torban darban keessaa guyyaa meeqaaf,sukkaara dhiiga kee yeroo ogeessi si gorse qoratte?	---------------lakkoofsa guyyootaa	

Kunuunsa Miilaa

T.lakk	Gaaffilee	Deebii tahuu dandahu	Yaada
Q410	Guyyoota torban darban keessaa guyyaa meeqaaf miila kee ilaalte?	---------------lakkoofsa guyyootaa	
Q411	Guyyoota torban darban keessaa guyyaa meeqaaf keessoo kophee keetii sakattaate?	---------------lakkoofsa guyyootaa	
Q412	Guyyoota torban darban keessaa guyyaa meeqaaf miila kee dhiqatte?	---------------lakkoofsa guyyootaa	
Q413	Guyyoota torban darban keessaa guyyaa meeqaaf miila kee jiiste?	---------------lakkoofsa guyyootaa	
Q414	Guyyoota torban darban keessaa guyyaa meeqaaf,erga dhiqattee booda quba miila kee gidduu gogsite?	---------------lakkoofsa guyyootaa	

Qoricha

T.lakk	Gaaffilee	Deebii tahuu dandahu	Yaada
Q415	Guyyoota torban darban keessaa guyyaa meeqaaf qoricha dhukkuba sukkaaraa si gorfame fudhatte?		
Q416	Guyyoota torban darban keessaa guyyaa meeqaaf qoricha insulini si gorfame fudhatte?		
Q417	Guyyoota torban darban keessaa guyyaa meeqaaf kiniinii dhukkuba sukkaaraa lakkoofsa si gorfame fudhatte?		

F. Tamboo Xuuxuu

T.lakk	Gaaffilee	Deebii tahuu dandahu	Yaada
Q418	Guyyoota torban darban keessaa guyyaa meeqaaf sigaaraa,paff illee xuuxxe?	Eeyyee<br>Lakki	

KUTAA SHAN: Of-kunuunsuu(Gorsa ogeessa fayyaa)

Bulchiinsa Nyaataa

kanneen armaan gadii keessaa gareen eegumsa fayyaa keessanii (Doctora,Nursii,)akka wantoota armaan gadii gootan isin gorsee jiraa?.

T.Lakk	Gaaffilee	Deebii tahuu dandahu		Yaada
		Eeyyee	lakki	
1.1	Karoora nyaata cooma xiqqaa qabu hordofaa			
1.2	Nyaata kaarbohydreetii walxaxaa hordofaa			
1.3	Ulfaatina qaamaa hir'isuuf baay'ina kaalorii nyaattu hir'isi			
1.4	Nyaata Fiberii baay'ee qabu nyaadhaa			

1.5	Furaaalee fi muduraalee yoo xiqqaate guyyaatti si'a shan nyaadhaa			
1.6	Mi'eessituu baay'ee muraasa nyaadhu(fkn nyaata mi'aawaa,karameellaa…)			
1.7	Gareen kunuunsa ogeessa fayyaa kee waa'ee nyaata kee irratti gorsa tokkollee siif hin kennine.			

SHAAKALA QAAMAA

Which of the following has your health-care team (doctor, nurse, dietitian, or diabetes educator) advised you to do? Please check all that apply.

T.lakk	Gaaffilee	Deebii tahuu kan dandahu		yaada
		Eeyyee	lakki	
2.1	Sochii qaamaa sadarkaa gad aanaa (kan akka deemsa guyyaa guyyaan gochuu)			
2.2	Sochii qaamaa walitti fufiinsa qabu daqiiqaa 20'f torbanitti si'a sadi taasisuu			
2.3	Sochii qaamaa ,hojii guyyaa guyyaa keessanitti karoorsaa(fkn karaa dheeraa deemuu,hojii humnaa hojjachuu ,liiftii osoo hin taane miila ofiin sadarkaa bahuu)			
2.4	Hamma,gosa,yeroo fi sadarkaa sochii qaamaa murtaa'e irratti bobba'uu			
2.5	Garee kunuunsa fayyaa kootiin waa'ee sochii qaamaa gorsi tokkollee hin kennamne			

Sadarkaa Sukkaaraa

Kanneen armaan gadii keessaa gareen eegumsa fayyaa keessan (doktorri,narsii,ogeessa nyaataa,kam akka gootan isin gorse?Kan isin ilaallatu hundaa Ilaalaa

T.Lakk	Gaaffilee	Deebii tahuu dandahu		Yaada
		Eeyyee	Lakki	
3.1	Dhiiga quba keessanii fi chaartii halluu fayyadadamuun sukkaara dhiiga keessanii qoradhaa			
3.2	Bu'aa isaa dubbisuuf maashinii fayyadamuun sukkaara dhiiga keessanii qoradhaa			
3.3	Fincaan keessan sukkaara qabaachuu isaa qoradhaa			

3.4	Garee kunuunsa Fayyaa kootiin waa'ee hamma sukkaara dhiiga koo ykn fincaan koo gorsi tokko illee hin kennamne			

Tamboo Xuuxuu

T.Lakk	Gaaffilee	Deebii tahuu dandahu		Yaada
		Eeyyee	Lakki	
4.1	Daawwannaa doktora yeroo dhumaaf goote irratti,haala tamboo xuuxuu kee namni gaafate jiraa?			
4.2	Yoo tamboo xuuxxe ,daawwannaa hakiima kee isa dhumaa irratti,waa'ee tamboo xuuxuu dhiisuu namni si gorse jiraa?			
4.3	Tamboo hin xuuxinaa (Gonkumaa).			
4.4	yeroo dhumaaf yoom sigaaraa xuuxxe?			
4.5	yeroo dhumaaf yoom sigaaraa xuuxxe?(waggaa lamaan dura.			
4.6	yeroo dhumaaf yoom sigaaraa xuuxxe?(Waggaa tokkoo hanga lamaa dura)			
4.7	yeroo dhumaaf yoom sigaaraa xuuxxe?(Ji'a Afurii hanga kudha lamaa dura)			
4.8	yeroo dhumaaf yoom sigaaraa xuuxxe?(Ji'a tokkoo hanga sadii dura)			
4.9	Yeroo dhumaaf yoom sigaaraa xuuxxe?Ji'a tokkoo hanga sadii			
4.10	yeroo dhumaaf yoom sigaaraa xuuxxe?(har'a)			

Gaaffilee xummurree Jirra heddu galatoomaa!!!
